# A Systematic Review on Cannabinoids for Neuropathic Pain Administered by Routes Other than Oral or Inhalation

**DOI:** 10.3390/plants11101357

**Published:** 2022-05-20

**Authors:** Jose-Manuel Quintero, German Pulido, Luis-Fernando Giraldo, Marta-Ximena Leon, Luis-Eduardo Diaz, Rosa-Helena Bustos

**Affiliations:** 1 Department of Clinical Pharmacology, Evidence-Based Therapeutics Group, Faculty of Medicine, Clínica Universidad de La Sabana, Universidad de La Sabana, Campus del Puente del Común, Km. 7, Autopista Norte de Bogotá, Chía 140013, Colombia; josequicas@unisabana.edu.co (J.-M.Q.); german.pulido@unisabana.edu.co (G.P.); 2 Doctoral Programme of Biosciences, Universidad de La Sabana, Chía 140013, Colombia; 3 Epidemiology and Biostatistics Department, School of Medicine, Universidad de La Sabana, Chía 140013, Colombia; luisf.giraldo@unisabana.edu.co; 4 Internal Medicine, Universidad de La Sabana, Chía 140013, Colombia; 5 Interventional Pulmonology and Research Department, Fundación Neumológica Colombiana, Bogotá D.C. 110131, Colombia; 6 Grupo Dolor y Cuidados Paliativos, Universidad de La Sabana, Chía 140013, Colombia; martha.leon@unisabana.edu.co; 7 Facultad de Ingeniería, Universidad de La Sabana, Campus del Puente del Común, Km. 7, Autopista Norte, Chía 140013, Colombia; luis.diaz1@unisabana.edu.co

**Keywords:** chronic pain, neuralgia, cannabinoids, drug administration routes

## Abstract

The use of *cannabis* and cannabinoid products for the treatment of neuropathic pain is a growing area of research. This type of pain has a high prevalence, limited response to available therapies and high social and economic costs. Systemic cannabinoid-based therapies have shown some unwanted side effects. Alternative routes of administration in the treatment of neuropathic pain may provide better acceptance for the treatment of multiple pathologies associated with neuropathic pain. To examine the efficacy, tolerability, and safety of cannabinoids (individualized formulations, phytocannabinoids, and synthetics) administered by routes other than oral or inhalation compared to placebo and/or conventional medications in the management of neuropathic pain. This systematic review of the literature reveals a lack of clinical research investigating cannabis by routes other than oral and inhalation as a potential treatment for neuropathic pain and highlights the need for further investigation with well-designed clinical trials. There is a significant lack of evidence indicating that cannabinoids administered by routes other than oral or inhaled may be an effective alternative, with better tolerance and safety in the treatment of neuropathic pain. Higher quality, long-term, randomized controlled trials are needed to examine whether cannabinoids administered by routes other than inhalation and oral routes may have a role in the treatment of neuropathic pain.

## 1. Introduction

Chronic neuropathic pain is associated with a significant health cost burden, as well as high societal costs [1,2,3]. The use of *cannabis* has been proposed in different diseases [4]. The endocannabinoid system is made up of endogenous cannabinoids, cannabinoid receptors, and the enzymes responsible for the synthesis and degradation of endocannabinoids. Cannabinoid receptors are the primary target for cannabinoids extracted from the cannabis plant for medical purposes known as phytocannabinoids [5,6]. Cannabinoids are used effectively in: control of nausea and vomiting in oncology, management of central spasticity, treatment of glaucoma, and pain relief in chronic diseases [7] as well as manage seizure syndromes [8].

Chronic neuropathic pain is associated with inflammation, mainly at the glial level. It can be started by a great variety of pathologies. The processes that are involved are under investigation and have not yet been completely elucidated. Neuropathic pain is recognized as a complex entity to treat [9]. The International Association for the Study of Pain (IASP) defines neuropathic pain as “pain caused by an injury or disease of the somatosensory nervous system” [10]. Neuropathic pain is a clinical description (and not a diagnosis) that requires a demonstrable injury or disease that meets established neurological diagnostic criteria [10] and it is a common entity [11]. The characteristics of neuropathic pain are different from those of other types of chronic pain [12]. Abnormal sensations such as allodynia, paresthesias, and dysesthesias are present and there are usually autonomic alterations [13]. Neuropathic pain partially responds to available treatments. A multimodal approach to this entity is recommended, but the available treatments have limitations. It is necessary to generate clinical evidence of new therapies to provide better therapeutic results [14,15].

Different treatment options have been described for neuropathic pain [15]. These include treatment with anticonvulsants, antidepressants, opioids, and local anesthetics [16]. There is no evidence to support the use of conventional analgesics such as paracetamol or non-steroidal anti-inflammatory drugs (NSAIDs) in the treatment of chronic neuropathic pain [16]. Some studies have addressed topical management with local anesthetics and capsaicin patches with results not always optimal [17,18,19,20,21]. The most widely accepted treatment is with neuromodulators, such as antidepressants (duloxetine and amitriptyline) [22] or anticonvulsants (gabapentin or pregabalin) [23]. On the other hand, the evidence for the efficacy of opioid use is low [24]. With these, the response is variable and side effects sometimes limit adherence to treatment. The approach with the best results is the multidisciplinary approach combining pharmacological, physical, and psychological interventions [25,26].

The endogenous cannabinoid system plays an important role in the regulation of homeostasis and neuroplasticity of the central nervous system (CNS), as well as in the modulation of pain transmission in the nociceptive pathway [27]. Cannabinoid receptors (CBR) are found throughout the entire CNS and peripheral, as well as in other organs [28]. CBR1 have been shown to be predominantly expressed on CNS neurons, while CBR2 are expressed on microglial cells that are activated in many neuroinflammatory diseases such as nerve-mediated pain. Neuropathic pain has complex pathophysiology, and its treatment can be challenging, making it a disease that is often not adequately treated in the clinical setting [29].

Systematic reviews of the use of cannabinoids for the treatment of chronic pain have been carried out [30]. There was moderate evidence to support cannabinoids in the treatment of chronic non-cancer pain. However, concerns regarding the adverse effects and safety of long-term cannabinoid use are still uncertain. There are recent studies [31], that suggest the use of new forms and administration vehicles of cannabinoid derivatives for the management of neuropathic pain, suppressing the possible adverse effects of their systemic administration. These new forms of cannabinoids administration could achieve a better pharmacokinetic (PK)/pharmacodynamic (PD) profile, which allows adequate adherence to these treatments, expanding the therapeutic possibilities and favoring clinical outcomes [32,33].

Possible benefits have been raised with cannabinoid derivatives in the management of neuropathic pain [34] and neuroinflammation [35]. The effects of cannabinoids and their interaction in the body are yet to be fully understood; however, the therapeutic effects of some cannabinoid derivatives are already approved for in the management of chronic pain and co-occurring conditions in some countries. The potential benefits of *cannabis*-based medicine (herbal *cannabis*, plant-derived or synthetic tetrahydrocannabinol (THC), THC/cannabidiol (CBD) oromucosal spray) in chronic neuropathic pain might be outweighed by their potential harms [36].

The endocannabinoid system is expressed and distributed in almost all human tissues [37], is activated by physiological stress and allows to control the state of balance. In the immune system, endocannabinoid signaling modulates the immune and inflammatory response in multiple states [38]. Preclinical studies demonstrate the beneficial effect of CBD treatment on autoimmune neuroinflammation by suppressing the expression of proinflammatory chemoattractants and regulating the activity of inflammatory macrophages [35]. Recent evidence shows that medical *cannabis* or cannabinoids result in little to very little improvement in pain relief, physical functioning, and quality of sleep among chronic pain patients [39]. However, this evidence was collected in chronic noncancer and cancer-related pain without establishing a specific route of administration or specifically identifying neuropathic pain.

Clinical trials with synthetic and natural *cannabis*-based drugs suggest a promising approach for the treatment of neuropathic pain of different etiologies [40,41]. However, adverse effects have been reported [42]. One way to control adverse effects in cannabinoid therapy is through a personalized medicine model [14]. Transdermal administration of cannabinoids may be a more effective alternative to the oral or inhaled route for the management of this challenging neuropathic pain condition. In a study in murine models, it was shown that the application of CBD in transdermal gel achieves a significant plasma concentration at steady state, suggesting the efficacy of this pathway administration [43]. It has been proposed topical and transdermal routes, seeking to obtain analgesic effect and reduce the systemic effects of oral and inhaled applications [32].

To date, there is no publication of a systematic review that addresses evidence on the safety and effectiveness of the use of cannabinoids other than the oral or inhaled route. Some patents have been registered [32]. Therefore, we aimed at evaluating the safety and effectiveness of cannabinoids used by routes other than oral or inhalation for neuropathic pain compared to placebo or other medications in terms of pain relief, quality of life and adverse events.

## 2. Results

**Figure 1:**
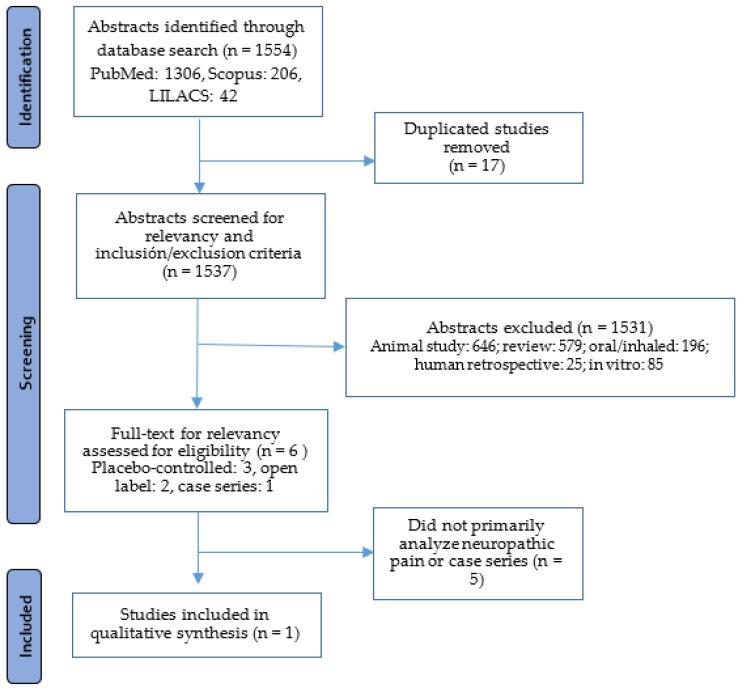
Flowchart of studies selected.

Using our search terms (Table S1), 1537 articles were selected. Of those, 196 were excluded because the route of administration was oral or inhaled, 646 were animal studies, 579 were reviews, 25 retrospective studies with oral or inhaled route of administration, and in vitro 85. Finally, 6 clinical studies in humans were reviewed in full text. These 6 studies were classified by study type as follows: 3 were placebo controlled, 2 open label, and 1 was a case series. Of the 6 human studies, only 1 was rated as “relevant” and completed the selection criteria as the data analyzed specifically included neuropathic pain, cannabinoid exposure, and the route of administration was other than oral and inhaled (Figure 1). Our results are briefly described below and summarized in Table 1.

Xu et al., recruited 29 patients with symptomatic peripheral neuropathy: 15 patients were randomized to the CBD group with the treatment product CBD-enriched emu oil containing 250 mg CBD/3 fl. oz, and 14 patients were randomized to the placebo group [31]. After four weeks, the placebo group was allowed to crossover into the treatment group for another four weeks. The neuropathic pain scale (NPS) was administered bi-weekly to assess the mean change from baseline to the end of the treatment period. The study population included 62.1% men and 37.9% women with a mean age of 68 years. Eighteen (62.1%) study subject participants had peripheral neuropathy secondary to diabetes mellitus, 6 (20.7%) participants had idiopathic peripheral neuropathy, and 3 (10.3%) participants had drug-related neuropathy. It additionally included one patient with embolism and one patient with sciatica [31]. There was a statistically significant decreasing trend (*p* < 0.05) in intense (in the CBD group decreased from 4.67 at baseline to 3.33 at week 4, difference: −1.34; in the placebo group decreased from 6.14 at baseline to 5.55 at week 4, difference: −0.59), sharp (in the CBD group decreased from 2.93 at baseline to 2.17 at week 4, difference: −0.76; in the placebo group decreased from 6.0 at baseline to 5.09 at week 4, difference: −0.91), cold (in the CBD group decreased from 2.13 at baseline to 0.5 at week 4, difference: −1.63; in the placebo group decreased from 2.79 at baseline to 2.36 at week 4, difference: −0.43) and itchy sensations (in the CBD group increased from 0.73 at baseline to 0.83 at week 4, difference: 0.1; in the placebo group decreased from 2.79 at baseline to 2.0 at week 4, difference: −0.79) in the CBD group compared to the placebo group. However, although statistically significant, the differences reported in the Table 2 of Xu et al. article [30] seems to favor the placebo over the CBD in the domains of sharp (CBD change at week 4 compared to baseline of −0.76 vs. a placebo change of −0.91) and itchy sensations (CBD change at week 4 compared to baseline of 0.1 vs. a placebo change of −0.79).

In particular, a greater reduction in intense, sharp, and itching sensations scores was observed. Furthermore, a significant time effect was also observed in the CBD group in reducing sharp, unpleasant, and surface pain ratings. No adverse events were reported in this study (Table 1) [31].

The risk of bias of the included study is summarized in Table 3. The randomization program was generated by a computer using blocks of size 4 and subjects were assigned accordingly; therefore, we rated the random sequence generation at low risk of bias. The risk of bias due to allocation concealment was judged to be high because the authors did not provide details about this point and in the baseline variables there were apparent imbalances between the CBD and placebo groups in the following variables: Gender, previous CBD use, Vibratory sensation, and NPS domains of Intense, Sharp, Itchy, Deep, Surface. Such imbalance implies a suboptimal effectivity of the randomization process, which could be related to ineffective allocation concealment. The study was double blind in its firsts phase, but it had a second phase which was open label and it is not clear how much the results of this second phase influenced some of the statistical analyses; therefore, the risk of bias related to blinding was rated as unclear. The study had 3 subjects lost to follow-up in each arm, indicating a lost to follow-up rate of about 20%, which is high and put the study at high risk of attrition bias. Finally, we rated the study at high risk of selective reporting bias because the study did not register the protocol before the beginning, making it impossible to know if the authors performed all the planned statistical tests, and when other common methods to assess the intervention effectivity, like assessing the change of NPS scores from baseline to week 4 (end of RCT blinded phase) are performed to the study data (published in a repository linked to the article), the benefits of the intervention are not confirmed.

Articles that were not included in qualitative synthesis for not meeting all the inclusion criteria are described below. Three articles studied the use of cannabinoid derivatives by routes other than oral and inhalation in neuropathic pain, but its design gave us serious concerns (Table 2). Participants had spinal cord injuries [44], facial posherpetic neuralgia [45] and back pain [46].

Hagenbach et al. evaluated the efficacy and side effects of oral D^9^-THC (THC) and rectal THC-hemisuccinate (THC-HS) in spinal cord injured patients [44]. It was planned as a three-phase study with crossover between oral THC (dronabinol), rectal THC, and placebo groups. The design was changed to two open-label phases with oral THC and rectal THC-HS, and finally, there was a random control trial of oral THC versus placebo. Hagenbach et al. found a significant reduction in the spasticity score (Ashworth scale) in patients treated with placebo. In total, 7 patients with THC-HS by rectal route were studied. Self-assessments of pain, mood, and attention were not reported for this group. However, one of these patients dropped out of the study due to pain [44]. In the oral group, phase 1 patients perceived a significant reduction in pain with oral THC on day 1 compared to baseline (*p* = 0.047). However, there was a trend (*p* = 0.066) for worse attention with oral THC compared with placebo on day 1 of treatment, but this trend disappeared despite continued treatment [44]. The open-label data collection for the THC treated group and departures from the planned analyses limits the conclusions that can be drawn, and for this reason it was excluded.

Phan et al. conducted an open-label trial without a placebo group, and this is the reason why it did not enter the final analysis. In total, 8 patients with facial postherpetic neuralgia received a cream containing the cannabinoid receptor agonist N-palmitoylethanolamine (PEA, Physiogel AI Creme^®^, Hamburg, Germany) [45]. The cream was applied to the affected site twice daily for two to four weeks. The course of symptoms was scored with the visual analog scale. In total, 5 of the 8 patients (62.5%) experienced a mean pain reduction of 87.8%. Therapy was tolerated by all patients. No unpleasant sensations or adverse events occurred [45].

Finally, Eskander et al. reported two patients that used topical CBD cream (400 mg CBD per two oz; Baskin Essentials Body Wellness Cream^®^) for the symptomatic relief of pain secondary to a lumbar compression fracture and in the mitigation of chest discomfort and dysesthesia secondary to a surgically resected meningioma, reporting significant symptom and pain relief [46]. This did not meet the inclusion criteria because it was a case report.

Two articles studied the use of cannabinoid derivatives by routes other than oral and inhalation in non-neuropathic pain. Because they do not focus on neuropathic pain, they are excluded. Jain et al. evaluated the use of intramuscular levonantradol versus placebo in acute postoperative pain [47]. Administration in a double-blind study of different single intramuscular doses of levonantradol (n = 40), an analog of the cannabinoid dronabinol, or placebo (n = 16) to 56 patients with moderate to severe postoperative or traumatic pain showed significant analgesic effects in comparison with placebo (*p* < 0.05) [47]. In total, 57 percent of patients managed with levonantradol reported as one or more of the side effects, whereas drowsiness was the most frequent. Changes in heart rate and blood pressure were also identified to be minor. Overall acceptability was good [47].

Schindler et al. conducted an exploratory, randomized, double-blind, placebo-controlled crossover study, which showed that psychoactive doses of intravenous delta-9-tetrahydrocannabinol failed to produce antinociceptive effects in healthy human volunteers (*n* = 6) [48]. Intravenous THC did not demonstrate significant antinociceptive properties in the experimental model of acute pain and capsaicin-induced hyperalgesia in healthy human subjects [48]. Schindler et al. conclude that continued study of THC and other cannabinoids through high-quality controlled studies in healthy volunteers and patients with pain conditions is warranted to inform the growing demand for the clinical application of cannabinoids in the treatment of pain [48] (Table 2).

## 3. Discussion

There are few good quality clinical studies evaluating the use of cannabinoids by routes of administration other than oral and inhaled. In the only study that met the inclusion criteria of our systematic review, there was a statistically significant reduction in intensity in the CBD group compared to the placebo group. In addition, a significant time effect was also observed. No adverse events were reported in this study.

In prospective trials and systematic reviews administered orally and inhaled, cannabinoids can cause some relief of neuropathic pain [30,36,49,50,51] in patients with neuropathic pain. There were no systematic reviews, like the present one, of studies in which cannabinoid derivatives are used by alternative routes to oral or inhaled. Only one study revealed by this search directly evaluated the relationship between topical *cannabis* and neuropathic pain. This demonstrates that there is a paucity of data on the possible risks and benefits of the use of cannabinoids by routes other than oral or inhaled to treat neuropathic pain.

There are many potential avenues for future research. The number of countries that have regulated the legal use, prescription, or sale of *cannabis* for medicinal purposes is increasing. Currently, more than 50 countries have adopted medical *cannabis* programs. This, together with the removal of the classification of *cannabis* as a Schedule I substance by the United Nations, allows progress in this field of research.

The clinical use of *cannabis* derivatives is already approved based on clinical evidence in entities such as spasticity in multiple sclerosis [52,53], seizures associated with two rare and severe forms of epilepsy, Lennox-Gastaut syndrome (LGS) [54,55] and Dravet syndrome (DS) [56,57], and cachexia-anorexia syndrome in cancer [58] and HIV [59]. There is a need for continued uniform evaluation of non-inhaled and non-oral *cannabis* use and neuropathic pain through rigorous, unbiased, and high-quality clinical research.

In the design of future research, multiple factors should be considered, including minimal clinically important difference, placebo-controlled studies, appropriate blinding protocols, and relevant outcome measures [60]. For trials of neuropathic pain, pain relief scales, patient and physician global impression of change, proportion of respondents (50% and 30% pain relief), validated neuropathic pain quality measures, and assessment of sleep, state mood, functional capacity, and quality of life have also been recommended [61], as well as functional outcomes. The lack of reliable epidemiological data has hampered progress in understanding the clinical impact of neuropathic pain and associated features [62]. Studies using the Leeds Assessment of Neuropathic Symptoms and Signs score (S-LANSS) [63], painDETECT [64], and Douleure Neuropathique en 4 questions (DN4) [65] indicate that standardized tools improve the quality of epidemiological data. Standardized tools for neuropathic pain may be useful in future trials because they could assess the efficacy of treatment for a specific symptom or combination of symptoms, rather than a disease entity [66].

The only selected study evaluated pain and specific sensations using the neuropathic pain scale (NPS). NPS was developed to assess the qualitative and quantitative qualities of neuropathic pain (NP) and has received prior validation in peripheral NP conditions [67]. The NPS appears to be able to discriminate between neuropathic and non-neuropathic pain. Whether diagnoses such as fibromyalgia and complex regional pain syndrome Type I can be classified as neuropathic is debated. Some studies of the NPS cutoff score suggest that these diagnoses may have a neuropathic pain component.

In addition, patients’ history of previous cannabinoid use, different routes of administration, and associated adverse events should be more closely examined.

Our review has several limitations. The databases consulted were limited to PubMed, SCOPUS, and LILACS. This could exclude articles from other databases that were not consulted. Additionally, the number of articles that met the inclusion criteria was limited. We found a great agreement in the selected articles, which could have produced selection bias.

The scarcity and diversity of studies regarding the subject and design of research in the area is evident and, therefore, the generalization of these results is limited. This further emphasizes the need for longitudinal studies that examine the potential risks and benefits of cannabinoid administration by alternative routes for neuropathic pain.

## 4. Materials and Methods

### 4.1. Search Eligibility Criteria and Search Strategy

This systematic review was developed with the recommendations given by the Cochrane collaboration [68]. We had a particular interest in studies related to the definitions of the Initiative on Methods, Measurement, and Pain Assessment in Clinical Trials (IMMPACT) group [69,70]. We focus on the use of *cannabis* derivatives by routes of administration other than oral and inhaled in the management of neuropathic pain by conducting a systematic review of the literature through various online databases. Data sources included PUBMED, SCOPUS, and LILACS. Search strategies used keywords placed in specific search fields (All fields and MeSH terms) on 4 April 2022 (Table S1).

Randomized clinical trials (RCTs), and reports of observational studies (with either a cohort design, case-series or a case–control design) that compared cannabinoids with usual care, placebo, or no treatment were eligible. We applied the following inclusion criteria: RCTs, cohorts, cases and controls that within their results reported relevant clinical outcomes in patients with neuropathic pain of any etiology, acute or chronic; age of participants: adults, 18 years or older; any publication year, language or status of publication (i.e., grey literature). We also applied the following exclusion criteria: Any study not presenting results against another treatment (including placebo or standard of care); studies with insufficient data for analysis; age of participants: younger than 18 years.

### 4.2. Selection of Studies, Data Extraction and Risk of Bias

All titles and abstracts identified in the electronic databases were screened independently of one another by two review authors (J.-M.Q., G.P.) determining eligibility by reading the abstract of each study identified by the search. We eliminated studies that clearly did not satisfy the inclusion criteria and obtained full copies of the remaining studies. Any discrepancies were resolved through a consensus discussion with a third senior reviewer (L.-F.G.).

Two review authors (J.-M.Q., G.P.) extracted data independently using a standard form and checked for agreement before selecting data, including information about the pain condition and number of participants treated, study setting, inclusion and exclusion criteria, demographic and clinical characteristics of the study samples and entering data into Review Manager 5.4 [71]. The two reviewers also independently assessed risk of bias for the included studies using the Cochrane Risk of Bias tool for randomised trial (RoB 2.0) and ROBINS-I 2016 tool for non-randomised studies.

### 4.3. Measures of Treatment Effect

For dichotomous data, we calculated the relative risk (RR), odds ratio (OR), inverse variance method and 95% Confidence Interval (CI). Continuous outcomes would be pooled using standardized mean differences and inverse variance method. In case of non-significant heterogeneity, the fixed-effect model would be used; otherwise, the random-effects model would be used. Results (mean difference, 95% CIs, and *p* values) from the between-group statistical analyses reported by the study were also extracted. The significance level was set at a *p* < 0.05 (two-tailed).

We consider a treatment response to achieve at least the minimally important difference (MID) [72]. The MID is the smallest amount of improvement in a treatment outcome that patients recognize as important [73]. In chronic neuropathic pain has not been determined. For the 10 cm VAS for pain and sleep quality, the MID has been established at approximately 1 cm [70,74]. Thus, we consider MID of 10% improvement on the scale of the analyzed study. We used Preferred Reporting Items for Systematic Review and Meta-Analysis PRISMA recommendations for the identification and selection of studies [75].

## 5. Conclusions

This systematic review of the literature revealed that there is a significant lack of evidence regarding the role of alternative oral and inhaled cannabinoid products in the treatment of neuropathic pain. It is not possible to determine the efficacy, tolerability, and safety of cannabinoids administered by those routes. Neuropathic pain is a clinical entity that is difficult to manage and is responsible for disability in many people with chronic pain. The clinical applications of cannabis and non-inhaled and non-oral cannabinoid products, including the management of neuropathic pain, clearly deserve further exploration. Higher quality, long-term, randomized controlled trials are needed to examine whether cannabinoids administered by routes other than inhalation and oral routes may have a role in the treatment of neuropathic pain.

## Figures and Tables

**Table 1:** Description of study selected.

Author	Design	Description	Gender	Age (yrs)	n	Outcome Pain Measures	Outcome Intervals	*p* Value Treatment Effect
Xu et al. (2020)	Single-centre, double-blind, randomized, placebo-controlled trial	Assess the efficacy of a topically delivered CBD oil in management of NP	M/F 18/11	35–79	29	Self-reported: pain and specific sensations were evaluated using the NPS in 10 domains of pain: sharp, hot, dull, cold, sensitive, itchy, deep and surface	Baseline, 2 and 4 weeks	Overall: 0.00901

**Table 2:** Detailed description of excluded studies after full text review.

Articles	Study Design	Description	Age (Years)	Size	Outcome Pain Measures	Outcome Intervals	Reason for Exclusion
Hagenbach et al. (2007)	Prospective	Assess the efficacy and side effects of oral D9-THC and rectal THC-HS in SCI patients, but the rectal arm was not performed	29–66	21	Self-reported: spasticity sum score using the MAS, self-ratings of VAS and spasticity	Baseline, 8 and 43 days	Did not assess routes other than oral or inhalation
Phan et al. (2009)	Prospective	Explores the analgesic efficacy of adjuvant therapy with a topical cannabinoid agonist in PHN patients with facial involvement	48–79	8	VAS	Baseline, 2 and 4 weeks	Did not include a control group
Eskander et al. (2020)	Retrospective	Describes the use of a hemp-derived CBD in a topical cream for the symptomatic relief in acute and chronic back pain	40–61	2	VAS	Baseline, 8 h and 4 weeks	Case report without a control group
Jain et al. (1981)	Prospective	Evaluation of intramuscular levonantradol and placebo in acute postoperative pain in patients with moderate to severe postoperative or trauma pain	25.3 ± 5 30.2 ± 11	56	Four point scale	Baseline, 15, 30, and 60 min, and hourly thereafter for a total of 6 h	Did not include patients with neuropathic pain
Schindler et al. (2019)	Prospective	Psychoactive doses of intravenous D9-THC in healthy volunteers induce chemical pain and hyperalgesia with capsaicin, mechanical (von Frey filament), hot and cold (thermode), and electrical (pulse generator)	19–51	6	VAS, MPQ-SF	Before drug administration, peak drug effects, and 2 h after drug administration	Study performed in healthy subjects, did not include patients with neuropathic pain

THC, tetrahydrocannabinol; THC-HS, THC-hemisuccinate; SCI, spinal cord injury; MAS, Modified Ashworth Scale; VAS, visual analogue scale; PHN, postherpetic neuralgia; CBD, cannabidiol.

**Table 3:** Cochrane Risk of Bias for Randomized Controlled Trials Tool for the study selected [31].

	Risk of Bias	Observations
Random sequence generation (selection bias)	Low	
Allocation concealment (selection bias)	High	The authors did not provide details about the method of allocation concealment and in the baseline variables there were apparent imbalance between the CBD and placebo groups in the following variables: Gender, previous CBD use, Vibratory Sensation, and NPS domains of Intense, Sharp, Itchy, Deep, Surface. Such imbalance could be due to deficiencies in the randomization process due to insufficient concealment
Blinding of participants and personnel (performance bias)	Unclear	The study had an open label phase, and it is not clear how much this influenced some of the statistical analyses
Blinding of outcomes assessment (detection bias)	Unclear	The study had an open label phase, and it is not clear how much this influenced some of the statistical analyses
Incomplete outcome data (attrition bias)	High	The lost to follow-up rate was high, about 20% (3 subjects in each arm)
Selective outcome reporting (reporting bias)	High	When other common methods to assess the intervention effectivity, like the change of NPS scores from baseline to week 4 (end of RCT blinded phase) are performed to the study data the benefits of the intervention are not confirmed and the protocol of the study was not published before its beginning

## Data Availability

Not applicable.

## Supplementary Materials

The following supporting information can be downloaded at: https://www.mdpi.com/article/10.3390/plants11101357/s1, Table S1: Search Strategy.

## References

[1] Meyers J.L., Madhwani S., Rausch D., Candrilli S.D., Krishnarajah G., Yan S. (2017). Analysis of real-world health care costs among immunocompetent patients aged 50 years or older with herpes zoster in the United States. Hum. Vaccines Immunother..

[2] Matthews S., De Maria A., Passamonti M., Ristori G., Loiacono I., Puggina A., Curran D. (2019). The Economic Burden and Impact on Quality of Life of Herpes Zoster and Postherpetic Neuralgia in Individuals Aged 50 Years or Older in Italy. Open Forum Infect. Dis..

[3] Yu S.-Y., Fan B.-F., Yang F., DiBonaventura M., Chen Y.-X., Li R.-Y., King-Concialdi K., Kudel I., Hlavacek P., Hopps M. (2019). Patient and economic burdens of postherpetic neuralgia in China. Clinicoeconomics Outcomes Res..

[4] Bridgeman M.B., Abazia D.T. (2017). Medicinal Cannabis: History, Pharmacology, And Implications for the Acute Care Setting. Pharm. Ther..

[5] Di Marzo V., Piscitelli F. (2015). The Endocannabinoid System and its Modulation by Phytocannabinoids. Neurotherapeutics.

[6] Mechoulam R., Parker L.A. (2013). The endocannabinoid system and the brain. Annu. Rev. Psychol..

[7] Allan G.M., Finley C.R., Ton J., Perry D., Ramji J., Crawford K., Lindblad A.J., Korownyk C., Kolber M.R. (2018). Systematic review of systematic reviews for medical cannabinoids: Pain, nausea and vomiting, spasticity, and harms. Can. Fam. Physician.

[8] Silvestro S., Mammana S., Cavalli E., Bramanti P., Mazzon E. (2019). Use of Cannabidiol in the Treatment of Epilepsy: Efficacy and Security in Clinical Trials. Molecules.

[9] Nicholson B., Verma S. (2004). Comorbidities in chronic neuropathic pain. Pain Med..

[10] IASP International Association for the Study of Pain Terminology. https://www.iasp-pain.org/resources/terminology/#neuropathic-pain.

[11] van Hecke O., Austin S.K., Khan R.A., Smith B.H., Torrance N. (2014). Neuropathic pain in the general population: A systematic review of epidemiological studies. Pain.

[12] Campbell J.N., Meyer R.A. (2006). Mechanisms of neuropathic pain. Neuron.

[13] Hansson P. (2002). Neuropathic pain: Clinical characteristics and diagnostic workup. Eur. J. Pain.

[14] Smith H.S., Sang C.N. (2002). The evolving nature of neuropathic pain: Individualizing treatment. Eur. J. Pain.

[15] Bates D., Schultheis B.C., Hanes M.C., Jolly S.M., Chakravarthy K.V., Deer T.R., Levy R.M., Hunter C.W. (2019). A Comprehensive Algorithm for Management of Neuropathic Pain. Pain Med..

[16] Moore R.A., Chi C.C., Wiffen P.J., Derry S., Rice A.S. (2015). Oral nonsteroidal anti-inflammatory drugs for neuropathic pain. Cochrane Database Syst. Rev..

[17] Derry S., Sven-Rice A., Cole P., Tan T., Moore R.A. (2013). Topical capsaicin (high concentration) for chronic neuropathic pain in adults. Cochrane Database Syst. Rev..

[18] Knezevic N.N., Tverdohleb T., Nikibin F., Knezevic I., Candido K.D. (2017). Management of chronic neuropathic pain with single and compounded topical analgesics. Pain Manag..

[19] Kocot-Kępska M., Zajączkowska R., Mika J., Kopsky D.J., Wordliczek J., Dobrogowski J., Przeklasa-Muszyńska A. (2021). Topical Treatments and Their Molecular/Cellular Mechanisms in Patients with Peripheral Neuropathic Pain-Narrative Review. Pharmaceutics.

[20] Rowbotham M.C., Davies P.S., Verkempinck C., Galer B.S. (1996). Lidocaine patch: Double-blind controlled study of a new treatment method for post-herpetic neuralgia. Pain.

[21] Sansone P., Passavanti M.B., Fiorelli A., Aurilio C., Colella U., De Nardis L., Donatiello V., Pota V., Pace M.C. (2017). Efficacy of the topical 5% lidocaine medicated plaster in the treatment of chronic post-thoracotomy neuropathic pain. Pain Manag..

[22] Lunn M.P., Hughes R.A., Wiffen P.J. (2014). Duloxetine for treating painful neuropathy, chronic pain or fibromyalgia. Cochrane Database Syst. Rev..

[23] Moore R.A., Straube S., Wiffen P.J., Derry S., McQuay H.J. (2009). Pregabalin for acute and chronic pain in adults. Cochrane Database Syst. Rev..

[24] Gaskell H., Derry S., Stannard C., Moore R.A. (2016). Oxycodone for neuropathic pain in adults. Cochrane Database Syst. Rev..

[25] Elmofty D.H., Anitescu M., Buvanendran A. (2013). Best practices in the treatment of neuropathic pain. Pain Manag..

[26] Cohen S.P., Vase L., Hooten W.M. (2021). Chronic pain: An update on burden, best practices, and new advances. Lancet.

[27] Cristino L., Bisogno T., Di Marzo V. (2020). Cannabinoids and the expanded endocannabinoid system in neurological disorders. Nat. Rev. Neurol..

[28] Mackie K. (2008). Cannabinoid receptors: Where they are and what they do. J. Neuroendocrinol..

[29] Colloca L., Ludman T., Bouhassira D., Baron R., Dickenson A.H., Yarnitsky D., Freeman R., Truini A., Attal N., Finnerup N.B. (2017). Neuropathic pain. Nat. Rev. Dis. Primers.

[30] Johal H., Devji T., Chang Y., Simone J., Vannabouathong C., Bhandari M. (2020). Cannabinoids in Chronic Non-Cancer Pain: A Systematic Review and Meta-Analysis. Clin. Med. Insights Arthritis Musculoskelet. Disord..

[31] Xu D.H., Cullen B.D., Tang M., Fang Y. (2020). The Effectiveness of Topical Cannabidiol Oil in Symptomatic Relief of Peripheral Neuropathy of the Lower Extremities. Curr. Pharm. Biotechnol..

[32] Stella B., Baratta F., Della Pepa C., Arpicco S., Gastaldi D., Dosio F. (2021). Cannabinoid Formulations and Delivery Systems: Current and Future Options to Treat Pain. Drugs.

[33] Maida V., Shi R.B., Fazzari F.G.T., Zomparelli L. (2020). Topical cannabis-based medicines—A novel paradigm and treatment for non-uremic calciphylaxis leg ulcers: An open label trial. Int. Wound J..

[34] Maayah Z.H., Takahara S., Ferdaoussi M., Dyck J.R.B. (2020). The molecular mechanisms that underpin the biological benefits of full-spectrum cannabis extract in the treatment of neuropathic pain and inflammation. Biochim. Biophys. Acta (BBA) Mol. Basis Dis..

[35] Dopkins N., Miranda K., Wilson K., Holloman B.L., Nagarkatti P., Nagarkatti M. (2021). Effects of Orally Administered Cannabidiol on Neuroinflammation and Intestinal Inflammation in the Attenuation of Experimental Autoimmune Encephalomyelitis. J. Neuroimmune Pharmacol..

[36] Mücke M., Phillips T., Radbruch L., Petzke F., Häuser W. (2018). Cannabis-based medicines for chronic neuropathic pain in adults. Cochrane Database Syst. Rev..

[37] Joshi N., Onaivi E.S. (2019). Endocannabinoid System Components: Overview and Tissue Distribution. Adv. Exp. Med. Biol..

[38] Almogi-Hazan O., Or R. (2020). Cannabis, the Endocannabinoid System and Immunity-the Journey from the Bedside to the Bench and Back. Int. J. Mol. Sci..

[39] Wang L., Hong P.J., May C., Rehman Y., Oparin Y., Hong C.J., Hong B.Y., AminiLari M., Gallo L., Kaushal A. (2021). Medical cannabis or cannabinoids for chronic non-cancer and cancer related pain: A systematic review and meta-analysis of randomised clinical trials. Bmj.

[40] Jensen B., Chen J., Furnish T., Wallace M. (2015). Medical Marijuana and Chronic Pain: A Review of Basic Science and Clinical Evidence. Curr. Pain Headache Rep..

[41] Nielsen S., Germanos R., Weier M., Pollard J., Degenhardt L., Hall W., Buckley N., Farrell M. (2018). The Use of Cannabis and Cannabinoids in Treating Symptoms of Multiple Sclerosis: A Systematic Review of Reviews. Curr. Neurol. Neurosci. Rep..

[42] Friedman D., French J.A., Maccarrone M. (2019). Safety, efficacy, and mechanisms of action of cannabinoids in neurological disorders. Lancet Neurol..

[43] Paudel K.S., Hammell D.C., Agu R.U., Valiveti S., Stinchcomb A.L. (2010). Cannabidiol bioavailability after nasal and transdermal application: Effect of permeation enhancers. Drug Dev. Ind. Pharm..

[44] Hagenbach U., Luz S., Ghafoor N., Berger J.M., Grotenhermen F., Brenneisen R., Mäder M. (2007). The treatment of spasticity with Delta9-tetrahydrocannabinol in persons with spinal cord injury. Spinal Cord.

[45] Phan N.Q., Siepmann D., Gralow I., Ständer S. (2010). Adjuvant topical therapy with a cannabinoid receptor agonist in facial postherpetic neuralgia. J. Dtsch. Dermatol. Ges..

[46] Eskander J.P., Spall J., Spall A., Shah R.V., Kaye A.D. (2020). Cannabidiol (CBD) as a treatment of acute and chronic back pain: A case series and literature review. J. Opioid Manag..

[47] Jain A.K., Ryan J.R., McMahon F.G., Smith G. (1981). Evaluation of intramuscular levonantradol and placebo in acute postoperative pain. J. Clin. Pharmacol..

[48] Schindler E.A.D., Schnakenberg Martin A.M., Sewell R.A., Ranganathan M., DeForest A., Pittman B.P., Perrino A., D’Souza D.C. (2020). In an exploratory randomized, double-blind, placebo-controlled, cross-over study, psychoactive doses of intravenous delta-9-tetrahydrocannabinol fail to produce antinociceptive effects in healthy human volunteers. Psychopharmacology.

[49] Boychuk D.G., Goddard G., Mauro G., Orellana M.F. (2015). The effectiveness of cannabinoids in the management of chronic nonmalignant neuropathic pain: A systematic review. J. Oral Facial Pain Headache.

[50] Meng H., Johnston B., Englesakis M., Moulin D.E., Bhatia A. (2017). Selective Cannabinoids for Chronic Neuropathic Pain: A Systematic Review and Meta-analysis. Anesth. Analg..

[51] Dykukha I., Malessa R., Essner U., Überall M.A. (2021). Nabiximols in Chronic Neuropathic Pain: A Meta-Analysis of Randomized Placebo-Controlled Trials. Pain Med..

[52] Vecchio D., Varrasi C., Virgilio E., Spagarino A., Naldi P., Cantello R. (2020). Cannabinoids in multiple sclerosis: A neurophysiological analysis. Acta Neurol. Scand..

[53] Markovà J., Essner U., Akmaz B., Marinelli M., Trompke C., Lentschat A., Vila C. (2019). Sativex^®^ as add-on therapy vs. further optimized first-line ANTispastics (SAVANT) in resistant multiple sclerosis spasticity: A double-blind, placebo-controlled randomised clinical trial. Int. J. Neurosci..

[54] Devinsky O., Patel A.D., Cross J.H., Villanueva V., Wirrell E.C., Privitera M., Greenwood S.M., Roberts C., Checketts D., VanLandingham K.E. (2018). Effect of Cannabidiol on Drop Seizures in the Lennox-Gastaut Syndrome. N. Engl. J. Med..

[55] Patel A.D., Mazurkiewicz-Bełdzińska M., Chin R.F., Gil-Nagel A., Gunning B., Halford J.J., Mitchell W., Scott Perry M., Thiele E.A., Weinstock A. (2021). Long-term safety and efficacy of add-on cannabidiol in patients with Lennox-Gastaut syndrome: Results of a long-term open-label extension trial. Epilepsia.

[56] Devinsky O., Patel A.D., Thiele E.A., Wong M.H., Appleton R., Harden C.L., Greenwood S., Morrison G., Sommerville K. (2018). Randomized, dose-ranging safety trial of cannabidiol in Dravet syndrome. Neurology.

[57] Miller I., Scheffer I.E., Gunning B., Sanchez-Carpintero R., Gil-Nagel A., Perry M.S., Saneto R.P., Checketts D., Dunayevich E., Knappertz V. (2020). Dose-Ranging Effect of Adjunctive Oral Cannabidiol vs Placebo on Convulsive Seizure Frequency in Dravet Syndrome: A Randomized Clinical Trial. JAMA Neurol..

[58] Smith L.A., Azariah F., Lavender V.T., Stoner N.S., Bettiol S. (2015). Cannabinoids for nausea and vomiting in adults with cancer receiving chemotherapy. Cochrane Database Syst. Rev..

[59] Badowski M.E., Yanful P.K. (2018). Dronabinol oral solution in the management of anorexia and weight loss in AIDS and cancer. Ther. Clin. Risk Manag..

[60] First L., Douglas W., Habibi B., Singh J.R., Sein M.T. (2020). Cannabis Use and Low-Back Pain: A Systematic Review. Cannabis Cannabinoid Res..

[61] Haanpää M., Attal N., Backonja M., Baron R., Bennett M., Bouhassira D., Cruccu G., Hansson P., Haythornthwaite J.A., Iannetti G.D. (2011). NeuPSIG guidelines on neuropathic pain assessment. Pain.

[62] Bennett M.I., Attal N., Backonja M.M., Baron R., Bouhassira D., Freynhagen R., Scholz J., Tölle T.R., Wittchen H.U., Jensen T.S. (2007). Using screening tools to identify neuropathic pain. Pain.

[63] Torrance N., Smith B.H., Bennett M.I., Lee A.J. (2006). The epidemiology of chronic pain of predominantly neuropathic origin. Results from a general population survey. J. Pain.

[64] Freynhagen R., Baron R., Gockel U., Tölle T.R. (2006). painDETECT: A new screening questionnaire to identify neuropathic components in patients with back pain. Curr. Med. Res. Opin..

[65] Bouhassira D., Attal N., Alchaar H., Boureau F., Brochet B., Bruxelle J., Cunin G., Fermanian J., Ginies P., Grun-Overdyking A. (2005). Comparison of pain syndromes associated with nervous or somatic lesions and development of a new neuropathic pain diagnostic questionnaire (DN4). Pain.

[66] Jensen M.P. (2006). Using pain quality assessment measures for selecting analgesic agents. Clin. J. Pain.

[67] Galer B.S., Jensen M.P. (1997). Development and preliminary validation of a pain measure specific to neuropathic pain: The Neuropathic Pain Scale. Neurology.

[68] Higgins J., Thomas J., Chandler J., Cumpston M., Li T., Page M.J., Welch V.A., Flemyng E. (2021). Cochrane Handbook for Systematic Reviews of Interventions Version 6.2.

[69] Dworkin R.H., Turk D.C., Peirce-Sandner S., Baron R., Bellamy N., Burke L.B., Chappell A., Chartier K., Cleeland C.S., Costello A. (2010). Research design considerations for confirmatory chronic pain clinical trials: IMMPACT recommendations. Pain.

[70] Dworkin R.H., Turk D.C., Wyrwich K.W., Beaton D., Cleeland C.S., Farrar J.T., Haythornthwaite J.A., Jensen M.P., Kerns R.D., Ader D.N. (2008). Interpreting the clinical importance of treatment outcomes in chronic pain clinical trials: IMMPACT recommendations. J. Pain.

[71] The Cochrane Collaboration (2020). Review Manager (RevMan).

[72] Busse J.W., Wang L., Kamaleldin M., Craigie S., Riva J.J., Montoya L., Mulla S.M., Lopes L.C., Vogel N., Chen E. (2018). Opioids for Chronic Noncancer Pain: A Systematic Review and Meta-analysis. JAMA.

[73] Schünemann H.J., Guyatt G.H. (2005). Commentary--goodbye M(C)ID! Hello MID, where do you come from?. Health Serv. Res..

[74] Zisapel N., Nir T. (2003). Determination of the minimal clinically significant difference on a patient visual analog sleep quality scale. J. Sleep Res..

[75] Liberati A., Altman D.G., Tetzlaff J., Mulrow C., Gøtzsche P.C., Ioannidis J.P., Clarke M., Devereaux P.J., Kleijnen J., Moher D. (2009). The PRISMA statement for reporting systematic reviews and meta-analyses of studies that evaluate healthcare interventions: Explanation and elaboration. Bmj.

