# Supplementary material for: A Systematic Review on Cannabinoids for Neuropathic Pain Administered by Routes Other than Oral or Inhalation
Source: Plants (Basel). 2022 May 20;11(10):1357. doi: 10.3390/plants11101357 (PMC9145866; doi:10.3390/plants11101357)
Supplement: Supplementary file 1 [file plants-11-01357-s001.zip › plants-1707562-supplementary.pdf]

Table S1. Search strategy

Database	Search Query	
**PubMed**	#1	(Pain AND (neuralgi* OR neuropathic)) OR Causalgia OR Facial Neuralgia OR Neuralgia OR Pain, Intractable OR Phantom Limb OR Reflex Sympathetic Dystrophy OR Complex Regional Pain Syndromes OR Acute Pain OR Chronic Pain OR Neuralgi* OR Neuropath*
	#2	Interventional AND pain OR Infusions, parenteral OR Infusions, intravenous OR Nerve Block OR Autonomic Nerve Block OR Cannabis OR Cannabinoids OR Medical Marijuana OR Therapeutic use OR Marijuana OR Marinol OR Dronabinol OR Nabilone OR Levonantradol OR Tetrahydrocannabinol OR Cesamet OR Delta-9-THC OR Delta-9-Tetrahydrocannabinol OR Nabiximols OR Sativex OR Cannabidiol OR THC OR CBD OR Cannab* OR Synthetic Drugs AND cannabinoids
	#3	Placebo OR Gabapentin OR Pregabalin OR Anesthetic OR Analgesics OR Drug therapy OR Imipramine OR Amytriptilin OR Carbamazepine OR Duloxetine OR Venlafaxine OR Lidocaine OR Bupivacaine OR Dexamethasone OR Methylprednisolone OR Drug therapy
	#4	Treatment Outcome OR (Visual Analog Scale AND pain) OR VAS Pain OR (Numeric scale AND pain) OR (Numerical scale AND pain) OR NRS Pain OR McGill Pain Questionnaire OR (MPQ AND pain) OR Short-Form McGill Pain Questionnaire OR (SF-MPQ AND pain) OR Chronic Pain Grade Scale OR (CPGS AND pain) OR Short Form-36 Bodily Pain Scale OR (SF-36 BPS AND pain) OR "Intermittent and Constant Osteoarthritis Pain" OR (ICOAP AND pain) OR "Faces Pain Scale-Revised" OR (FPS-R AND pain) OR Pain intensity OR pain severity
	#5	Emotional states OR Profile Mood States OR (POMS AND mood) OR Center Epidemiologic Studies Depression Scale OR (CES-D AND depression) OR Geriatric Depression Scale OR (GDS AND depression) OR (Hospital Anxiety Depression Scale) OR (HADS AND depression) OR Patient Health Questionnaire-9 OR PHQ-9 OR State-Trait Anxiety Inventory OR (STAI AND anxiety) OR Beck Depression Inventory-II (BDI-II AND beck) OR Treatment Outcome OR (Visual Analog Scale AND pain) OR VAS Pain OR (Numeric scale AND pain) OR (Numerical scale AND pain) OR NRS Pain OR McGill Pain Questionnaire OR (MPQ AND pain) OR Short-Form McGill Pain Questionnaire OR (SF-MPQ AND pain) OR Chronic Pain Grade Scale OR (CPGS AND pain) OR Short Form-36 Bodily Pain Scale OR (SF-36 BPS AND pain) OR ("Intermittent and Constant Osteoarthritis Pain") OR (ICOAP AND pain) OR "Faces Pain Scale-Revised" OR (FPS-R AND pain) OR Pain intensity OR pain severity
	#6	Patient Reported Outcome Measures OR Patient Outcome Assessment OR Patient participation OR Patient-reported outcome OR Measures questionnaire development procedures OR Physical functioning OR Health-related quality of life OR HRQOL OR Multidimensional Pain Inventory OR (MPI AND pain) OR Interference Scale OR Brief Pain Inventory OR (BPI AND pain) OR Pain interference OR General activity
	#7	Adverse effects OR Drug-Related Side Effects and Adverse Reactions OR Injection site reaction OR Adverse events
	#8	Efficacy OR Effectiveness OR Medication Therapy management
	#9	((Efficacy OR Effectiveness OR Medication Therapy management OR (Adverse effects OR Drug-Related Side Effects Adverse Reactions) OR Injection site reaction OR Adverse events OR Treatment outcome OR PGIC OR Patient Global Impression Change scale OR Patient Reported Outcome Measures OR Patient Outcome Assessment OR Patient participation OR Patient-reported outcome OR Measures questionnaire development procedures OR Physical functioning OR Health-related quality life OR HRQOL OR Multidimensional Pain Inventory OR (MPI AND pain) OR Interference Scale OR Brief Pain Inventory OR (BPI AND pain) OR Pain interference OR General activity) OR (Emotional states OR Profile Mood States OR (POMS AND mood) OR Center Epidemiologic Studies Depression Scale OR (CES-D AND depression) OR Geriatric Depression Scale OR (GDS AND depression) OR (Hospital Anxiety Depression Scale) OR (HADS AND depression) OR Patient Health Questionnaire-9 OR PHQ-9 OR State-Trait Anxiety Inventory OR (STAI AND anxiety) OR Beck Depression Inventory-II (BDI-II AND beck) OR Treatment Outcome OR (Visual Analog Scale AND pain) OR VAS Pain OR (Numeric scale AND pain) OR (Numerical scale AND pain) OR NRS Pain OR McGill Pain Questionnaire OR (MPQ AND pain) OR Short-Form McGill Pain Questionnaire OR (SF-MPQ AND pain) OR Chronic Pain Grade Scale OR (CPGS AND pain) OR Short Form-36 Bodily Pain Scale OR (SF-36 BPS AND pain) OR ("Intermittent and Constant Osteoarthritis Pain") OR (ICOAP AND pain) OR "Faces Pain Scale-Revised" OR (FPS-R AND pain) OR Pain intensity OR pain severity)
	#11	#1 AND #2 AND #3 AND (#4 OR #5 OR #6 OR #7 OR #9)

**SCOPUS**	("Visual Analog Scale" AND pain) OR (VAS AND Pain) OR ("Numeric scale" AND pain) OR (NRS AND Pain) OR "McGill Pain Questionnaire" OR (MPQ AND pain) OR ("SF-MPQ" AND pain) OR (Chronic Pain Grade Scale) OR (CPGS AND pain) OR (Short Form-36 Bodily Pain Scale) OR (SF-36 BPS) OR (Osteoarthritis AND Pain) OR (ICOAP AND pain) OR (Faces Pain Scale-Revised) OR (FPS-R) OR (Pain intensity) OR (pain severity) OR (Treatment Outcome) AND Cannabis OR Cannabinoids OR (Medical Marijuana) OR (Marijuana AND "Therapeutic use") OR Marijuana OR Marinol OR Dronabinol OR Nabilone OR Levonantradol OR Tetrahydrocannabinol OR Cesamet OR "Delta-9-THC" OR "Delta-9-Tetrahydrocannabinol" OR Nabiximols OR Sativex OR Cannabidiol OR THC OR CBD OR (Synthetic Drugs AND cannabinoids) AND<br>(efficacy OR effectiveness OR medication AND therapy AND management) AND<br>(pain OR causalgia OR facial AND neuralgia OR neuralgia OR pain, OR intractable<br>OR phantom OR limb OR reflex AND sympathetic AND dystrophy OR complex AND regional AND pain AND syndromes OR acute AND pain OR chronic AND pain)
**LILACS**	("Visual Analog Scale" AND pain) OR (VAS AND Pain) OR ("Numeric scale" AND pain) OR (NRS AND Pain) OR "McGill Pain Questionnaire" OR (MPQ AND pain) OR ("SF-MPQ" AND pain) OR (Chronic Pain Grade Scale) OR (CPGS AND pain) OR (Short Form-36 Bodily Pain Scale) OR (SF-36 BPS) OR (Osteoarthritis AND Pain) OR (ICOAP AND pain) OR (Faces Pain Scale-Revised) OR (FPS-R) OR (Pain intensity) OR (pain severity) OR (Treatment Outcome) AND Cannabis OR Cannabinoids OR (Medical Marijuana) OR (Marijuana AND "Therapeutic use") OR Marijuana OR Marinol OR Dronabinol OR Nabilone OR Levonantradol OR Tetrahydrocannabinol OR Cesamet OR "Delta-9-THC" OR "Delta-9-Tetrahydrocannabinol" OR Nabiximols OR Sativex OR Cannabidiol OR THC OR CBD OR (Synthetic Drugs AND cannabinoids) AND ("Visual Analog Scale" AND pain) OR (VAS AND Pain) OR ("Numeric scale" AND pain) OR (NRS AND Pain) OR "McGill Pain Questionnaire" OR (MPQ AND pain) OR ("SF-MPQ" AND pain) OR (Chronic Pain Grade Scale) OR (CPGS AND pain) OR (Short Form-36 Bodily Pain Scale) OR (SF-36 BPS) OR (Osteoarthritis AND Pain) OR (ICOAP AND pain) OR (Faces Pain Scale-Revised) OR (FPS-R) OR (Pain intensity) OR (pain severity) OR (Treatment Outcome) AND Cannabis OR Cannabinoids OR (Medical Marijuana) OR (Marijuana AND "Therapeutic use") OR Marijuana OR Marinol OR Dronabinol OR Nabilone OR Levonantradol OR Tetrahydrocannabinol OR Cesamet OR "Delta-9-THC" OR "Delta-9-Tetrahydrocannabinol" OR Nabiximols OR Sativex OR Cannabidiol OR THC OR CBD OR (Synthetic Drugs AND cannabinoids)
